# Histopathology of a retrocorneal membrane after Descemet membrane endothelial keratoplasty: a case report

**DOI:** 10.1186/s13256-018-1960-0

**Published:** 2019-02-06

**Authors:** Tarek Bayyoud, Jens Martin Rohrbach, Karl Ulrich Bartz-Schmidt, Sebastian Thaler

**Affiliations:** Department of Ophthalmology, University Hospital of Tübingen, Elfriede-Aulhorn-Str. 7, 72076 Tübingen, Germany

**Keywords:** Retrocorneal membrane, Descemet’s membrane endothelial keratoplasty, Graft failure, Histopathology

## Abstract

**Background:**

We report the first histopathologically proven occurrence of a retrocorneal membrane after Descemet’s membrane endothelial keratoplasty.

**Case presentation:**

A white Caucasian 76-year-old woman received penetrating keratoplasty on her right eye 2 years after Descemet’s membrane endothelial keratoplasty surgery with combined cataract extraction and intraocular lens implantation for Fuchs’ endothelial corneal dystrophy due to an allograft rejection with ensuing graft failure.

Her preoperative vision was counting fingers (20/2000) caused by immunological debris, corneal edema, and secondary membrane formation. Her postoperative vision at 3 months was 20/125.

The histopathological evaluation showed a membranous structure overlying the denuded Descemet membrane.

**Conclusions:**

We report a case of a histopathologically proven retrocorneal membrane after Descemet’s membrane endothelial keratoplasty surgery.

## Introduction

Descemet’s membrane endothelial keratoplasty (DMEK) is a method to transplant donor corneal endothelium with its adjacent Descemet’s membrane (DM) to replace respective diseased recipient tissues [1, 2]. Fuchs’ endothelial corneal dystrophy (FECD) is the most common indication besides bullous keratopathy and other secondary endothelial decompensations. The short-to-mid-term visual outcomes were excellent achieving 20/25 or even better visual acuities in a large proportion of operated patients [3, 4]. However, potential complications exist in terms of graft preparation, graft implantation, and postoperative follow-up [5]. One of the most common postoperative complications represents graft detachment. Allograft rejection and graft failure are significantly less common, but may still demand re-DMEK or penetrating keratoplasty [6]. Thus far, retrocorneal membrane formation was reported once in the literature following DMEK but without histopathological correlate [7]. After many different types of intraocular surgeries, membranous structures may ensue. It is well known that these structures can penetrate keratoplasty and it is therefore not a novelty in itself [8].

## Case presentation

A white Caucasian 76-year-old woman visited our tertiary referral center with the complaint of decreased vision in both eyes. Clinically bilateral corneal guttae were evident with corneal bullae on her right eye (OD). She was diagnosed as having bilateral FECD subjectively worse on her OD and a DMEK was advised. Her preoperative visual acuity was 20/40 OD and left eye (OS).

### Preparation

After staining the donor endothelium with trypan blue 0.06% for 30 seconds, an 8.0-mm graft was dissected using the forceps’ technique according to Melles immediately prior to surgery.

### Transplantation

After standard cataract extraction with a 2.75-mm limbal tunnel incision and two 1-mm incisions at 10 and 2 o’clock, viscoelastic was removed by extensive irrigation/aspiration. The descemetorhexis was performed under air using a price hook (Moria S.A. plc, 92160 Antony, France) and the diseased tissue removed with a stromal scraper.

A standard no-touch technique was applied to keep iatrogenic endothelial trauma to a minimum. The stained DMEK graft was inserted into the anterior chamber using a custom-made glass injector, oriented and adhered onto the recipient’s stroma using air pressurization.

Postoperatively the graft was attached, no further intervention was needed, and no immunological reactions were noted. A standard postoperative regimen was followed (moxifloxacin eye drops four times a day for 2 weeks and prednisolone eye drops four times a day with slow tapering). Her postoperative visual acuity was 20/50 with significant subjective improvement (uncorrected with persistent stromal haze).

**Fig. 1:**
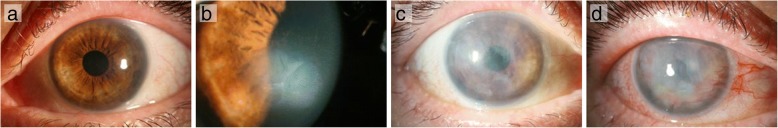
**a** After Descemet’s membrane endothelial keratoplasty. **b** Prior to Descemet’s membrane endothelial keratoplasty. **c** After the acute rejection episode. **d** During the acute rejection. (1) Deep neovascularizations beyond the 8.0-mm-descemetorhexis; (2) profound stromal haze; (3) membranous, sheet-like structures expanding along the posterior corneal surface (not observable on all images)

After 18 months she returned with decreased vision and an allograft rejection. During the acute episode a pronounced, conjunctival injection, corneal edema, and neovascularizations were prominent. Superficial and deep neovascularizations beyond the 8.0-mm-descemetothexis were observed. The cornea itself had signs of a non-functioning graft with increased corneal thickness, extensive edema, and endothelial cell attenuation on specular microscopy. In addition, a stromal haze and retrocorneal membranous structures were visualized on slit-lamp microscopy (Fig. 1a–d).

Although local steroids were intensified, the retrocorneal membranes persisted and the graft eventually failed completely. The retrocorneal structures were thin, mesh-like, and whitish in color. A penetrating keratoplasty was advised and the removed tissue sent for histopathological evaluation. On morphological examination, the retrocorneal membranes had an undulating character with an adjacent bare DM with no to very scarce endothelial remnants.

**Fig. 2:**
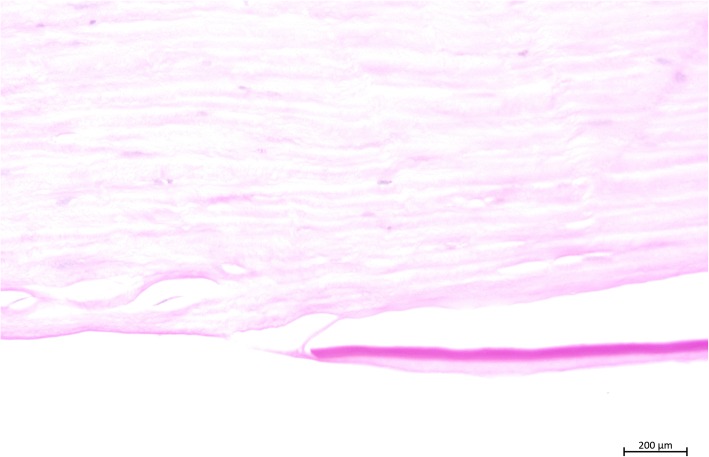
Histological examination (hematoxylin and eosin stain). (1) Atrophic or a denuded endothelial layer at the “wound margin” is present; (2) in addition, a disrupted Descemet’s membrane with a gap is observed (not observable on slit-lamp examination); (3) the fibrous structure originates from the corneal stroma, traverses the Descemet’s membrane gap, and expands onto the bare Descemet’s membrane

The histopathological report stated an endothelial insufficiency secondary to a retrocorneal fibrous membrane and deep neovascularizations secondary to an allograft rejection (Fig. 2).

**Fig. 3:**
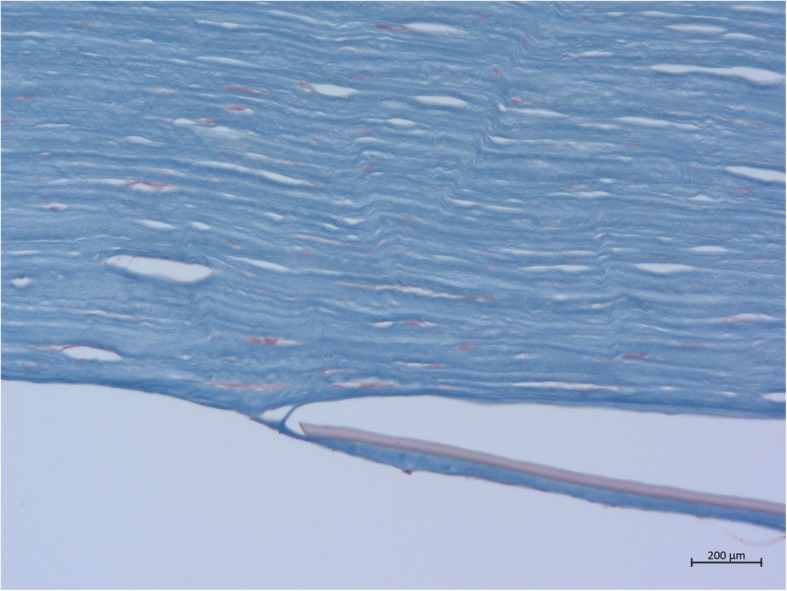
Masson staining for connective tissue. The transplanted Descemet membrane turns reddish

From the posterior stroma a thin membrane of connective tissue/corneal stroma had grown on the back of the lamellar graft. This membrane continued to the right so that more than 50% of the graft was covered, eventually leading to endothelial decompensation. On histological examination, it was an “ordinary retrocorneal membrane,” as is often observed after penetrating keratoplasty. After DMEK, however, such a membrane has not previously been described histologically (Fig. 3).

## Discussion

Postoperative complications after DMEK include, among others, graft detachment, graft rejection, and graft failure. By far the most common of these represent graft detachments of which, in turn, the majority is of minor extent. Retrocorneal membrane formation is a much rarer complication and thoroughly studied after penetrating keratoplasty [8]. The etiology of retrocorneal membranes rests in one of three mechanisms:

1. epithelial downgrowth/ingrowth;
2. keratocytic downgrowth;
3. fibrous metaplasia of the corneal endothelium.

After traumatic or iatrogenic injury, corneal wound healing may follow two distinct pathways. The first is a regenerative one and is characterized by endothelial cell enlargements, cell migration, and formation of a continuous cell layer. The second pathway has features of cell proliferation, collagen production, and loss of contact inhibition. This leads to the formation of a fibrotic non-regenerative tissue with contractile aspects. Some authors further discriminate between inflammatory and non-inflammatory types of membranes [9].

We wanted to present a case of retrocorneal membrane formation after DMEK with histopathological correlate. In this case, irreversible transplant failure occurred despite immunosuppression with intensified local steroids every hour necessitating a penetrating keratoplasty. Histopathological examination revealed the presence of a retrocorneal membrane. The membrane formation was associated with an irreversible graft failure.

In comparison to other retrocorneal membranes, for example, as after failed penetrating keratoplasties, the following features were in common:

1. character of the membrane (diffuse, retrocorneal, and fibrous);
2. timing of retrocorneal membrane formation;
3. endothelial cell loss.

The retrocorneal membrane after DMEK in this case was of the non-regenerative fibrotic type with contractile aspects. This was implicated by the histopathological recipient–donor interface exhibiting a membranous structure in direct contact with the corneal stroma prior to extension over the bare Descemet membrane of the graft. The endothelial cell loss was complete and substantial; it was exhibited clinically by corneal decompensation. Histopathology showed no remaining endothelial cells. The current case was clinically characterized by stromal involvement with haze implicating no functional endothelial cell regeneration.

Thus, in this case, we did not observe an endothelial-mesenchymal transformation. A transformation may be a potential therapeutic target, if the origin of the retrocorneal membrane is the corneal endothelium itself [10].

In general, these membranous structures consist of thin sheets of fibrous tissue. The etiology is supposed to be a fibroblastic or stromal downgrowth, fibrous metaplasia of the corneal endothelium, or a combination of the two [11, 12]. According to Kremer *et al*., three pathophysiological conditions are required: first, the capability to regenerate scar tissue; second, a gap in Descemet’s layer; and third, atrophic endothelium at the wound margins [8]. All three conditions were met (Fig. 2). The membranous structure has its origin at the recipient–donor interface. At this junction the fibroblastic or stromal downgrowth and/or fibrous metaplasia of the corneal endothelium may be sought. The recipient–donor interface as the origin has also been observed in other studies [13, 14].

The first observation of such a membrane was in 1901 by Fuchs [15]. In general, retrocorneal fibrous membranes may be related to irreversible transplant failures as in the case described [8].

## Conclusions

Retrocorneal membranes may occur not only after penetrating keratoplasty but also after DMEK. To the best of our knowledge, we have described such a membrane for the first time histopathologically after DMEK. Graft failure was associated in this case with the formation of the pathological membrane.

## Abbreviations

DM: Descemet’s membrane
DMEK: Descemet membrane endothelial keratoplasty
FECD: Fuchs’ endothelial corneal dystrophy
OD: Right eye
OS: Left eye
